# Stromal Cell-Derived Factor 1 Protects Brain Vascular Endothelial Cells from Radiation-Induced Brain Damage

**DOI:** 10.3390/cells8101230

**Published:** 2019-10-10

**Authors:** Jong-Ik Heo, Kwang Il Kim, Sang-Keun Woo, Joong Sun Kim, Kyu Jin Choi, Hae-June Lee, Kwang Seok Kim

**Affiliations:** 1 Divisions of Radiation Biomedical Research, Korea Institute of Radiological and Medical Sciences, Seoul 01812, Korea; talester@nate.com (J.-I.H.); ichoco@kirams.re.kr (K.J.C.); hjlee@kirams.re.kr (H.-J.L.); 2 School of Radiological and Medico-Oncological Sciences, University of Science and Technology, Daejeon 34054, Korea; 3 Divisions of Radio-Isotope Applied Research, Korea Institute of Radiological and Medical Sciences, Seoul 01812, Korea; kikim@kirams.re.kr (K.I.K.); skwoo@kirams.re.kr (S.-K.W.); 4 K-herb Research Center, Korea Institute of Oriental Medicine, Daejeon 34054, Korea; centraline@kiom.re.kr

**Keywords:** SDF-1, CXCR4, ionizing radiation, endothelial dysfunction, senescence, brain disorder

## Abstract

Stromal cell-derived factor 1 (SDF-1) and its main receptor, CXC chemokine receptor 4 (CXCR4), play a critical role in endothelial cell function regulation during cardiogenesis, angiogenesis, and reendothelialization after injury. The expression of CXCR4 and SDF-1 in brain endothelial cells decreases due to ionizing radiation treatment and aging. SDF-1 protein treatment in the senescent and radiation-damaged cells reduced several senescence phenotypes, such as decreased cell proliferation, upregulated p53 and p21 expression, and increased senescence-associated beta-galactosidase (SA-β-gal) activity, through CXCR4-dependent signaling. By inhibiting extracellular signal-regulated kinase (ERK) and signal transducer and activator of transcription protein 3 (STAT3), we confirmed that activation of both is important in recovery by SDF-1-related mechanisms. A CXCR4 agonist, ATI2341, protected brain endothelial cells from radiation-induced damage. In irradiation-damaged tissue, ATI2341 treatment inhibited cell death in the villi of the small intestine and decreased SA-β-gal activity in arterial tissue. An ischemic injury experiment revealed no decrease in blood flow by irradiation in ATI2341-administrated mice. ATI2341 treatment specifically affected CXCR4 action in mouse brain vessels and partially restored normal cognitive ability in irradiated mice. These results demonstrate that SDF-1 and ATI2341 may offer potential therapeutic approaches to recover tissues damaged during chemotherapy or radiotherapy, particularly by protecting vascular endothelial cells.

## 1. Introduction

With an increase in the worldwide elderly population, there is an increasing awareness of age-related impairments in cognitive function that present significant individual and socioeconomic impacts. Recent studies suggest that radiation exposure, even at low doses, could trigger cognitive dysfunctions seen in normal aging and lead to the development of Alzheimer’s disease [1,2]. Recent reports demonstrate in vivo evidence that radiation-induced normal tissue injuries, particularly intestinal injuries, are caused by endothelium-dependent mechanisms [3]. It is also possible that the altered regulation of fundamental aging mechanisms may contribute to the pathogenesis of neurodegenerative disorders [4]. Although central nervous system aging and diverse triggers of neurodegenerative processes are still the focus of cognitive decline and disease studies and treatments, the possible contribution of cerebrovascular deficiencies has been recently revealed [5]. Endothelial cell loss causes blood vessel leakage that can lead to cognitive decline [6,7]. Since cerebromicrovascular endothelial cells are a key component of the blood–brain barrier, damage to capillaries, especially in the cerebral cortex and hippocampus, causes regional cerebral blood flow decreases, reduced cerebral glucose utilization, the loss of vascular innervation, and eventually cognitive failure [5,8,9,10].

In addition, as brain aging and the resulting cognitive impairments are the results of multiple interacting cellular and molecular events, there is a need for additional investigation into the interactions of neuroendocrine, inflammatory, and microvascular mechanisms of cognitive impairments. Therefore, the microvasculature and its circulating factors, including key nutrients, trophic factors, and substrates, that are necessary for normal function, must be understood in cognitive pathology development. Recent reports of parabiosis experiments established that a circulating factor in young mouse blood improves the brain function of more mature mice [11,12]. Particularly, growth differentiation factor 1 has been identified as a positive regulator of neurogenesis, cerebral vasculature, neuronal activity, and cognitive function in the hippocampus of old mice [13,14].

Here, we focused on the circulating factor stromal cell-derived factor 1 (SDF-1). SDF-1 and its main receptor, CXC chemokine receptor 4 (CXCR4), are chemokine/chemokine receptor pairs that have various functions in a cellular environment. CXCR4 is expressed in endothelial, smooth muscle, and endothelial progenitor cells and promotes angiogenesis both in vitro and in vivo [15,16]. Activation of CXCR4 by SDF-1 modulates various biological processes, such as cell chemotaxis, proliferation, apoptosis, survival, and differentiation [17]. In addition, CXCR4/SDF1 promotes cardiomyocyte survival and neovascularization and plays an effective role in tissue regeneration after myocardial infarction and vascular disease [18]. Moreover, CXCR4/SDF-1 restores signs of typical acute radiation syndromes, such as gastrointestinal syndrome, but the exact pathobiology of neurovascular disorders is not clear.

Recent reports have confirmed that CXCR4 and CXCR receptor type 7 activates serine/threonine-specific protein kinase (AKT), mitogen-activated protein kinase (MAPK), and Janus kinase/signal transducer and activator of transcription protein 3 (JAK/STAT3) [19,20,21]. Furthermore, recent studies suggest that circulating factors can regulate vascular function as well as improve memory and cognitive function through insulin-like growth factor-1 [22], MAPK-ERK [23], and Sma- and Mad-related protein 2/3 (SMAD2/3) signaling [24]. Moreover, ERK and STAT3 are known to promote angiogenesis by regulating the integrin signaling cascade, and the activation of ERK and STAT3 increases angiogenesis by increasing vascular endothelial growth factor expression. Hence, we aimed to determine whether signaling through MAPK or JAK/STAT3 is associated with SDF-1-induced vasculature and brain function improvements.

In the present study, we examined whether CXCR4/SDF-1 affects recovery from radiation-induced endothelial cell damage and senescence. Additionally, we investigated the role of CXCR4/SDF-1 in tissue damage and cognitive ability changes that may be caused by deoxyribonucleic acid (DNA) damage in vivo. Although the molecular mechanisms of radiation-induced cognitive decline are not fully known, understanding both the biological effects of ionizing radiation (IR) and SDF-1 function in cerebromicrovascular endothelial cells can help develop therapies to prevent cognitive disorders with aging and improve cognitive function after brain damage.

## 2. Materials and Methods

### 2.1. Cell Culture and Animals

Human brain microvascular endothelial cells (HBMVECs) were purchased from iXcells Biotechnologies (San Diego, CA, USA), cultured in Endothelial Growth Media-2 Microvascular Medium (Lonza, Walkersville, MD, USA) culture medium, and incubated in an incubator at 37 °C in a 5% carbon dioxide humidified incubator. When the cells reached 80% confluence, trypsinization and passaging were performed. The proliferation rate was determined by calculating the number of population doublings (PDs). For experiments, HBMVECs were used in either passage 6 (PD < 28) or passage 12 (PD > 50). These are referred to as young and old cells in this study, respectively. For IR treatment, cells were exposed to γ-rays from a 137 Cs γ-ray source (Atomic Energy of Canada, Ltd., Chalk River, ON, Canada) administered at a dose of 3.5 Gy/min. To assess the damage caused by radiation, cells were treated with 1 μM/mL Doxorubicin (Sigma, St. Louis, MO, USA), an anticancer drug, and the media was changed after 4 h. To inhibit cell damage, HBMVECs were treated with 50 ng/mL of a recombinant SDF-1 protein (ProSpec Ltd., East Brunswick, NJ, USA) and 1 μg/mL of a CXCR4 agonist, ATI2341 (Tocris Bioscience, Bristol, UK).

For in vivo experiments, 7-week-old female C57BL/6 and BALB/c nude mice were obtained from Orient Biotech Co., Ltd. (Gyeonggi-do, Korea). Mice were allowed access to a normal diet and autoclaved water ad libitum and housed in a pathogen-free facility. All mouse procedures were approved by the Institutional Animal Care and Use Committee (IACUC) of the Korea Institute of Radiological and Medical Sciences (IACUC permit number: KIRAMS2017-0013).

### 2.2. Transfection of CXCR4 siRNA

ON-TARGETplus siRNAs (Dharmacon Inc., Lafayette, CO, USA) specific for CXCR4 were transfected to HBMVECs using the Lipofectamine 2000 transfection reagent (Life Techonologies Inc., Gaithersburg, MD, USA) according to the manufacturer’s suggestion. siRNAs of CXCR4 were transfected at 50 nM/mL. As negative control, off-target control, ON-TARGETplus Non-targeting Control siRNA (Dharmacon Inc., Lafayette, Co, USA), was used and treated at a concentration of 50 nM/mL. After transfection for 8 h, the media were replaced.

### 2.3. Ribonucleic Acid Extraction and Real-Time Polymerase Chain Reaction Analysis

Ribonucleic acid (RNA) was prepared from cells using a TRIsure solution (Bioline, London, UK) according to the manufacturer’s instructions. Complementary DNA was synthesized using a sensiFASTTM complimentary DNA Synthesis kit (Bioline, London, UK) with the isolated RNA. Real-time polymerase chain reaction (PCR) was performed using specific primers (Table 1) with a qualitative PCR SYBR 2X Master Mix kit (Mbiotech, Gyeonggi-do, Korea) and the LightCycler 96 Real-Time PCR system (Roche Diagnostic Corp., IN, USA). All primers were designed. Glyceraldehyde-3-Phosphate Dehydrogenase (GAPDH) was used to normalize the qPCR transcript levels. Primer design, target specificity, and PCR efficiency, according to Minimum Information for Publication of Quantitative Real-Time PCR Experiments (MIQE) guidelines, were verified. Target specific primer design using primer blast and bioinformatics tool, single peak was confirmed in the melting curve, single band was confirmed by gel electrophoresis, and the short product size of 70 to 150 bp was confirmed.

### 2.4. Western Blotting

Western blotting experiments were performed by following a previous study [25]. The primary antibodies for CXCR4 (Enzo Life Sciences, Farmingdale, NY, USA), phospho-p53, STAT3, phosphor-ERK1/2, ERK1/2, phosphor-Rb (Cell Signaling Technology, Danver, MA, USA), p53, p21, phosphor-STAT3, Nibrin (NBS1), breast cancer type 1 susceptibility protein (BRCA1), checkpoint kinase 1 (Chk1), Chk2, Cyclin A (Santa Cruz, CA, USA), zonula occludens-1 (Zo-1), occludin (OCLN) (Thermo Scientific, Waltham, MT, USA), and Claudin-5 (MILLPORE, Damstadt, DE, USA) were used. The concentration of antibodies used was 1:1000 for primary antibodies and 1:2000 for secondary antibodies. After reacting with the antibody, the protein was observed by chemiluminescence using Amersham Imager 600 (GE Healthcare, Chicago, IL, USA); beta-actin was used as the loading control.

### 2.5. Enzyme-Linked Immunosorbent Assay

Supernatants of cell cultures were collected for measurement of secretion of SDF-1 by specific enzyme-linked immunosorbent assay (ELISA) (LifeSpan Biosciences, Seattle, WA, USA) according to the manufacturer’s protocol.

### 2.6. Cell Growth Assay

Cells were cultured in 60-mm dishes. After sub-culturing, cells were cultured in 96-well plates and 6-well plates, according to the date, and the absorbance was measured spectrophotometrically at 570 nm using a 1.25 mg/mL 3-(4,5-dimethylthiazol-2-yl)-2,5-diphenyltetrazolium bromide (MTT) solution (Sigma, St. Louis, MO, USA). Cells were detached and counted to confirm cell proliferation.

### 2.7. Senescence-Associated Beta-Galactosidase Staining

Senescence-associated beta-galactosidase staining experiments were performed by following a previous study [25].

### 2.8. Tube Formation Assay

Forty thousand cells were seeded on a Matrigel- (Corning Life Sciences, Lowell, MA, USA) coated 12-well plate and incubated for 8 h. Tube formation was observed by an optical microscope. Tube length was measured by ImageJ software (National Institute of Health, Bethesda, MD, USA).

### 2.9. γH2AX Foci Staining

Two thousand cells were seeded on a 10-hole slide and incubated for 24 h. The cells were treated with radiation (4 Gy) and allowed to react for 1, 3, or 6 h. Cells were fixed with 4% paraformaldehyde and blocked with 5% donkey serum (Sigma, St. Louis, MO, USA) at room temperature for 1 h. The antibody for the phosphorylated form of H2A histone family member X (γH2AX (Cell signaling Technology, Danvers, MA, USA)), was incubated overnight at 4 °C. Cells were incubated with Alexa Fluor 488-conjugated goat anti-mouse IgG (Life Technologies Corporation, Carlsbad, CA, USA) and 4′,6-diamidino-2-phenylindole (DAPI). After mounting with Vectashield H-1200 (Vector Laboratories, Burlingame, CA, USA), the number of γH2AX foci was measured using a confocal microscope (LSM 900, Carl Zeiss, Oberkochen, Germany).

### 2.10. Radiation and Treatment with CXCR4 Agonist ATI2341

Animals were anesthetized by an intraperitoneal injection of tiletamine/zolazepam (Zoletil 50^®^; Virak Korea; Seoul, Korea) and exposed to cranial irradiation (whole-brain included) using an X-RAD 320 system (Precision X-ray, Inc., North Branford, CT, USA) at 250 kV and 10 mA with 420 mm of aluminum shielding, resulting in a dose rate of 2 Gy/min. Sham-irradiated mice were anesthetized and immobilized for the same period without radiation. ATI2341 (Tocris Bioscience, Bristol, UK) was dissolved in 2.5% dimethyl sulfoxide/phosphate buffer solution and was intraperitoneally administered to the mice 1 h before radiation exposure at a dose of 10 mg/kg. Control mice received an identical vehicle dosage in a similar manner.

### 2.11. Transendothelial Electrical Resistance and Permeability Assay

The insert well was coated with collagen in an incubator for 30 min. One million cells were cultured in a 24-well transwell plate (Corning Life Sciences, Lowell, MA, USA) for 72 h until monolayer formation. The endothelial electrical resistance of the cells was measured using a Millipore Millicell ERS-2 system (Millipore Corporation, Billerica, MA, USA). The electrical resistance was calculated from the background-corrected values. The permeability assay was performed using Fluorescein isothiocyanate (FITC)-dextran (Sigma, St. Louis, MO, USA). Basal medium was added to the bottom well, and 1 mg/mL FITC-dextran was added to the upper well. After 15 min of incubation, FITC-dextran (excitation maximum 485 nm; emission maximum 530 nm) was measured with the media of the bottom well using a spectrophotometer (Multiskan FC, Thermo Fisher Scientific, Waltham, MA, USA).

### 2.12. TUNEL Assay in the Small Intestine

Whether ATI2341 could affect radiation-induced gastrointestinal damage was investigated after systemic radiation of the mice with 8 Gy. The small intestine of BALB/c nude mice was injected with ATI2341 (intraperitoneal, 10 mg/kg) 1 h before 8 Gy of radiation. The small intestine was dissected 3 days later and cryosectioned for terminal deoxynucleotidyl transferase dUTP nick end labeling (TUNEL) staining. Apoptotic cells were stained by catalytically incorporating fluorescein-12dUTP at 3′-OH DNA ends using recombinant terminal deoxynucleotidyl transferase. The experiment was performed using a DeadEndTM Fluorometric TUNEL kit (Promega, Fitchburg, WI, USA) according to the manufacturer’s methods.

### 2.13. Brain Blood Vessel Isolation

Cerebral blood vessels of irradiated and ATI2341-treated mice were isolated according to Boulay’s purification protocol [26]

### 2.14. Blood Flow Analysis

Seven-week-old female BALB/c nude mice were irradiated locally on the left leg with 8 Gy. ATI2341 (intraperitoneal, 10 mg/kg) was injected 1 h before irradiation. After 3 days, optical imaging was performed in real-time as soon as the fluorescent dye indocyanine green (Sigma, St. Louis, MO, USA) was injected in the tail-vein using a MaestroTM in vivo fluorescence imaging system (Cambridge Research & Instrumentation, Woburn, MA, USA). The average fluorescence signal of each region of interest (ROI) over the left leg was measured and recorded. Results were expressed as vessel-to-background ratios.

### 2.15. Novel Object Recognition Memory Test

The novel object recognition test was assessed to evaluate recognition memory after radiation-induced brain injury. For experiment adaptation, animals were handled for 10 min daily for 7 consecutive days before the test. Mice were randomly assigned to 4 groups (*n* = 6); (1) sham (vehicle) control, (2) ATI2341, (3) IR, and (4) both ATI2341 and IR. For another 3 days, mice were habituated to the experimental conditions; mice were acclimated to the testing box (width, 45 cm; length, 45 cm; and height, 30 cm) without objects for 10 min daily. Then, animals received ATI2341 administration and/or IR according to a schedule. For instance, radiation (5 Gy) was applied locally to the head, and the experiment was performed after 24 h. At a training session, 2 identical objects were placed 15 cm apart in an object recognition testing apparatus. Mice were allowed to explore the objects in the apparatus for 10 min. At a testing session, 1 of the objects was located once more in the same way as the training session, and the other was replaced with a new, differently shaped (novel) object. The animals moved around freely in the object recognition testing box for 10 min. Mouse activity and exploration behavior were recorded during training and testing sessions. Behavior was recorded on video, and the exploration time and visit number for each object were measured by a video analysis program (Viewer3, BIOSERVE GmbH, Mainz, Germany). We considered that if a mouse retained the memory of a previously encountered object, it would show a preference for the novel object; the percentage preference was defined as the number of interactions with a specific object divided by the total number of interactions with both objects. After behavioral testing, mice were euthanized following an IACUC-approved approach, and each hemibrain was extracted for histological and molecular analysis. One hemibrain of each mouse was fixed in 4% paraformaldehyde/phosphate buffer solution; the other hemibrain of each mouse was dissected, and the hippocampus was immediately placed on ice as described previously [27] and stored at −80 °C for Western blotting or qualitative PCR. 

### 2.16. Statistical Analysis

The results are expressed as means ± standard deviations. The differences between the groups were compared by the unpaired *t*-test or 1-way analysis of variance (ANOVA) using GraphPad Prism (GraphPad software, La Jolla, CA, USA). The behavioral data and protein expression data were analyzed by 2-way ANOVAs using GraphPad Prism. All data are presented as the mean ± standard error of the mean. Significance was defined as *p* < 0.05.

## 3. Results

### 3.1. Decline of CXCR4 and SDF-1 Expression with IR Treatment and Aging in Brain Endothelial Cells

**Figure 1:**
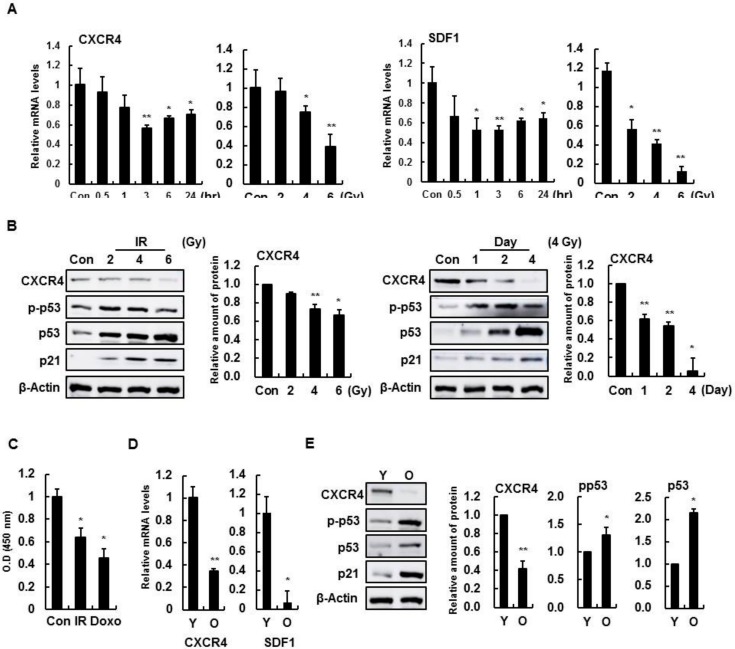
Induction of cell damage in human brain microvascular endothelial cells (HBMVECs) reduces CXC chemokine receptor 4 (CXCR4) and stromal cell-derived factor 1 (SDF-1). (**A**) HBMVECs were treated with IR (4 Gy) for 0, 0.5, 1, or 3 h with increasing ionizing radiation (IR) concentrations for 24 h. *CXCR4* and *SDF-1* expression levels were measured using real-time polymerase chain reaction (PCR). (**B**) Protein levels of CXCR4, phospho-p53, p53, and p21 were confirmed in radiation-treated HBMVECs with the indicated antibodies in a Western blot analysis. (**C**) HBMVECs were treated with 4 Gy radiation or 1 μM/mL doxorubicin for 24 h, and the secreted SDF-1 protein level was measured with cell supernatants using an enzyme-linked immunosorbent assay (ELISA) analysis. (**D**) Expression levels of *CXCR4* and *SDF-1* were determined by real-time PCR in senescent HBMVECs. (**E**) Protein levels of CXCR4 were also confirmed by Western blot analysis in senescent HBMVECs. Values are expressed as the mean ± standard deviation of three independent experiments. * *p* < 0.05 and ** *p* < 0.01. IR, ionizing radiation; Doxo, doxorubicin; Y, young cell; O, old cell; Con, control; CXCR4, CXC chemokine receptor 4; SDF-1, stromal cell-derived factor 1; HBMVEC, human brain microvascular endothelial cell.

To determine whether CXCR4 and SDF-1 expression were altered with IR treatment, expression was confirmed by dose- and time-dependent radiation changes in HBMVECs. The expression of CXCR4 and SDF-1 decreased, and molecules related to cell cycle arrest, such as p53 and p21, increased as the radiation time and dose increased (Figure 1A,B). Likewise, CXCR4 and SDF-1 expression were also decreased in more aged HBMVECs (Figure 1C,D). These results demonstrate that CXCR4 and SDF-1 expression is involved in cellular senescence and radiation-induced damage in brain endothelial cells.

### 3.2. Effect of SDF-1 on Senescent HBMVECs and IR Exposure

**Figure 2:**
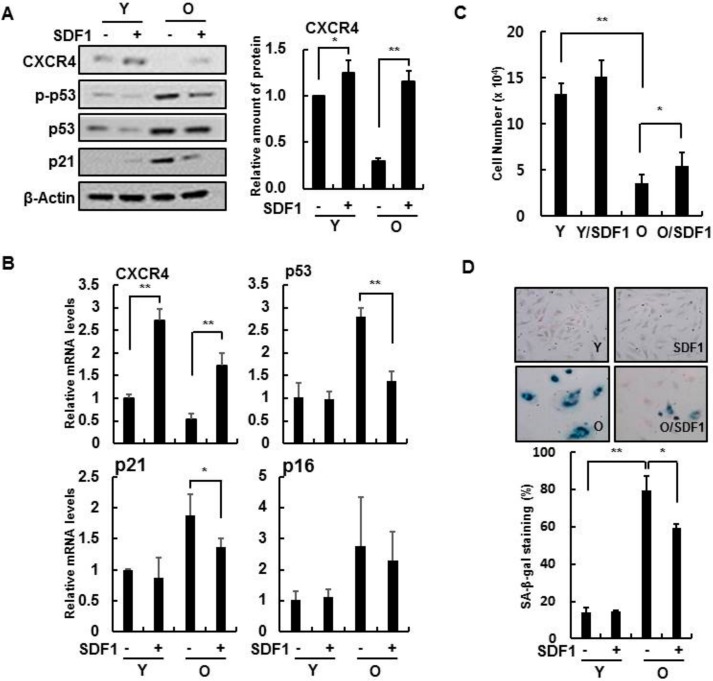
SDF-1 induces CXCR4 expression in senescent HBMVECs. (**A**) Young and old HBMVECs were treated with 50 ng/mL recombinant SDF-1 protein for 24 h. Protein levels of CXCR4, phospho-p53, p53, and p21 were determined via Western blotting. (**B**) In each cell, expression levels of *CXCR4, p53, p21,* and *p16* were measured using real-time polymerase chain reaction analysis. (**C**) To measure cell proliferation, the cells were treated with 50 ng/mL SDF-1 for 4 days, and the number of cells was counted. (**D**) Cells were stained for SA-β-gal activity and evaluated using a light microscope (100×). senescence-associated beta-galactosidase (SA-β-gal) positive cells were analyzed and expressed as a percentage. Values are expressed as the mean ± standard deviation of three independent experiments. * *p* < 0.05 and ** *p* < 0.01. Y, young cells; O, old cells.

In senescent HBMVECs, treatment with recombinant human SDF-1 protein increased CXCR4 expression while decreasing p21 expression and p53 activity, which are senescence markers (Figure 2A,B). In addition, exogenous SDF-1 reduced several cellular senescence phenotypes, such as increased cell proliferation (Figure 2C) and SA-β-gal activity (Figure 2D), suggesting that SDF-1 can partially reverse cellular senescence in brain endothelial cells. 

**Figure 3:**
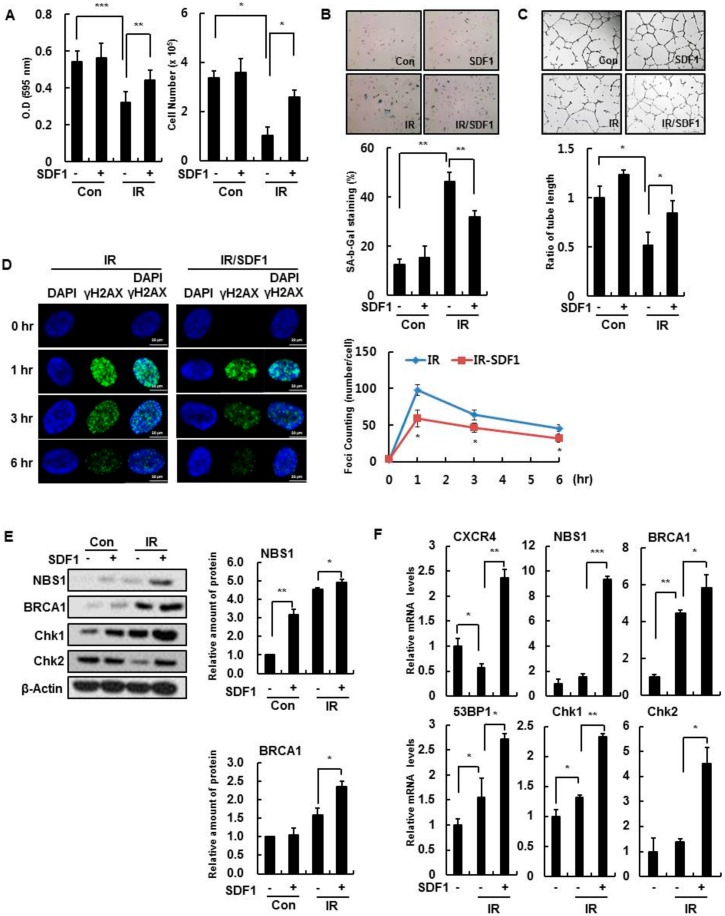
SDF-1 promotes recovery in damaged HBMVECs. (**A**) Cell viability was measured by a 3-(4,5-dimethylthiazol-2-yl)-2,5-diphenyltetrazolium bromide (MTT) assay, and cell proliferation was confirmed by cell count analysis. HBMVECs were pretreated with 50 ng/mL SDF-1 an hour before 4 Gy radiation treatment. MTT was measured 24 h after radiation treatment, and the cell count was analyzed after 4 days. (**B**) Cells were stained for SA-β-gal activity and evaluated using a light microscope (100×). Four days after irradiation, staining was performed to analyze SA-β-gal positive cells; results are expressed as a percentage. (**C**) Cells were pretreated with SDF-1 an hour before irradiation for 24 h. Cells were placed on Matrigel, and tube formation was measured after incubating for 8 h. (**D**) HBMVECs were treated with SDF-1 for an hour, irradiated for 0, 1, 3, or 6 h. Cells were immunostained with a γH2AX antibody to measure foci in more than 100 cells; scale bars = 20 μm. (**E**) Proteins were isolated 24 h after irradiation, and levels of DNA damage response-related proteins were measured via Western blot analysis. (**F**) Expression levels of DNA damage response-related genes were measured by real-time polymerase chain reaction analysis. Values are expressed as the mean ± standard deviation of three independent experiments. * *p* < 0.05, ** *p* < 0.01, and *** *p* < 0.001. Con, control; IR, ionizing radiation; IR/SDF1, SDF-1, and radiation treated group; γH2AX, phosphorylated H2A histone family member X; NBS1, Nibrin; BRCA1, Breast cancer type 1 susceptibility protein; Chk, checkpoint kinase.

Likewise, we investigated whether SDF-1 affected several cellular phenotypes during IR exposure. Recombinant human SDF-1 treatment partially restored cell proliferation (Figure 3A), inhibited SA-β-gal activity (Figure 3B), and recovered tube forming ability in IR-damaged endothelial cells (Figure 3C). Furthermore, SDF-1 reduced the number of γH2AX foci per nucleus, a marker of DNA damage, that was present in IR-treated cells (Figure 3D), indicating that SDF-1 affects the functioning of the DNA repair system after IR exposure. SDF-1 induced the expression of repair system-associated genes, such as *NBS1, 53BP1, BRCA1 and Chk1*, after IR treatment (Figure 3E,F). Therefore, SDF-1 treatment partially restores endothelial cell function deficits in IR-damaged and senescent HBMVECs.

### 3.3. SDF-1 Plays a Role in a CXCR4-Dependent Mechanism

**Figure 4:**
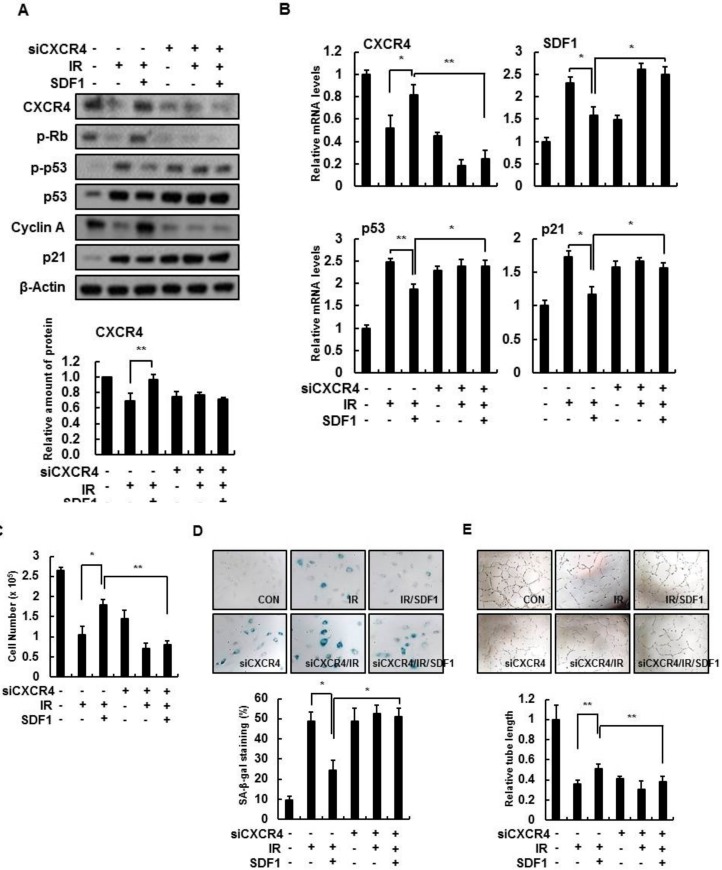
CXCR4 is important for SDF-1 function. (**A**) HBMVECs were transfected with CXCR4 siRNA (50 nM/mL). Western blot analysis was conducted in radiation (4 Gy)- and SDF-1 (50 ng/mL)-treated cells with CXCR4 deletions. (**B**) Expression levels of *CXCR4, SDF-1, p53,* and *p21* were measured using real-time polymerase chain reaction analysis. (**C**) Cell number analysis was performed to measure cell proliferation. (**D**) SA-β-gal staining (×100) was performed in siCXCR4-transfected cells. SA-β-gal positive cells were measured and expressed as a percentage. (**E**) Tube formation experiments were performed by SDF-1 treatment in CXCR4-depleted cells. Values are expressed as the mean ± standard deviation of three independent experiments. * *p* < 0.05 and ** *p* < 0.01. siRNA, scrambled siRNA-transfected group; siCXCR4, CXCR4 siRNA-transfected group; IR, ionizing radiation; IR/SDF1, SDF-1, and radiation treated group.

To assess whether SDF-1 action depends on CXCR4 signaling, the effect of SDF-1 on IR-induced senescence of HBMVECs was confirmed after deleting CXCR4. By Western blot analysis, SDF-1 effects, such as increased expression of CXCR4, phospho-Rb, and cyclin A, were not present, nor was a decrease in expression of phospho-p53, p53, and p21 in CXCR4-deleted cells (Figure 4A). Based on real-time PCR, SDF-1 during IR did not affect CXCR4-deficient cells (Figure 4B). In IR-treated HBMVECs, increased cell proliferation by SDF-1 treatment was not shown (Figure 4C); in addition, SA-β-gal activity was not decreased by SDF-1 treatment in CXCR4-deficient cells (Figure 4D). Furthermore, CXCR4-deficient cells did not increase tube-forming ability even with SDF-1 treatment (Figure 4E). Thus, although radiation-induced cellular senescence is inhibited by SDF-1 treatment, SDF-1 effects depend on CXCR4 activation.

### 3.4. Effect of A CXCR4 Agonist on HBMVECs

**Figure 5:**
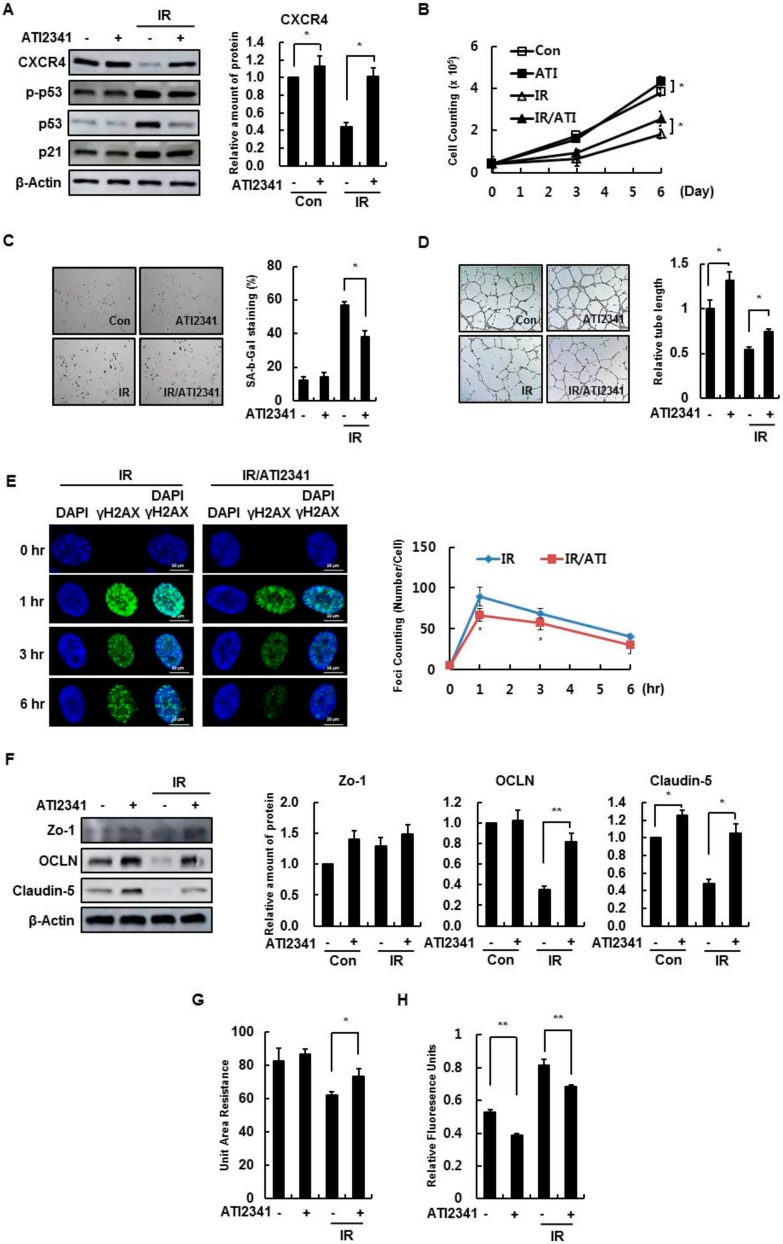
A CXCR4 agonist, ATI2341, enhances the cellular function of HBMVECs. (**A**) HBMVECs were irradiated (4 Gy) after 1 μg/mL ATI2341 treatment and incubated for 24 h. Levels of CXCR4, p53, and p21 proteins were measured via Western blotting. (**B**) Cell proliferation was measured by cell counting. Cells were plated at 4 × 10^4^ and cultured for 3 or 6 days, and the cells were counted. (**C**) HBMVECs were treated with 1 μg/mL ATI2341 and/or 4 Gy radiation and incubated for 4 days. The number of SA-β-gal positive cells were measured. (**D**) The tube lengths of HBMVECs treated with ATI2341 and/or radiation (4 Gy) and seeded on Matrigel were measured and compared to controls. (**E**) HBMVECs were treated with ATI2341 for 1 h, then irradiated (4 Gy) for 0, 1, 3, or 6 h. Cells were immunostained with a γH2AX antibody, and the number of foci was measured; scale bars = 20 μm. (**F**) By Western blot, tight junction proteins (Zo-1, OCLN, and Claudin-5) were analyzed in ATI2341- and/or radiation (4 Gy)-treated cells. (**G**) ATI2341 and/or radiation (4 Gy) treated HBMVECs were cultured on a 24-well Transwell plate (5 × 10^5^) to measure the transendothelial electrical resistance values that were measured after a cell monolayer formed, 72 h after irradiation. (**H**) A permeability assay was performed to observe leakage from 5 × 10^5^ ATI2341- and radiation (4 Gy)-treated HBMVECs that were cultured in a 24-well Transwell plate and maintained for 72 h. The upper well was treated with Dextran (10 μg/150 μL) for 15 min, and relative fluorescence units in the lower well media were measured. Values are expressed as the mean ± standard deviation of three independent experiments. * *p* < 0.05 and ** *p* < 0.01. Con, control; ATI, a CXCR4 agonist.

The agonist ATI2341 was examined as a potentially viable alternative to inhibiting endothelial cell damage by CXCR4/SDF-1 signaling. The effective concentration was confirmed in the cell proliferation experiment through treatment by concentration (Figure S1). Agonist treatment suppressed increases in phospho-p53 and p21 caused by radiation (Figure 5A). In addition, radiation-induced cell proliferation decreases were restored by ATI2341 treatment (Figure 5B). Radiation-induced cell senescence also increased SA-β-gal activity; this effect was decreased by ATI2341 (Figure 5C). Moreover, the decline in capillary tube formation by radiation damage was restored to ATI2341 treatment (Figure 5D), and ATI2341 reduced radiation-induced formations of γH2AX foci (Figure 5E). 

The expression of tight junction proteins, such as Zo-1, OCLN, and Claudin-5, decreased in irradiated HBMVECs, based on the Western blot data, and the reduced number of tight junction proteins was restored by AIT2341 treatment (Figure 5F). Treating HBMVECs with ATI2341 reduced monolayer leakage caused by radiation, based on an increase in the radiation-reduced transendothelial electrical resistance value (Figure 5G), and a decrease in dextran diffusion by ATI2341 according to the permeability measurements (Figure 5H). These results indicate that the activation of CXCR4 by ATI2341 promotes the recovery of radiation-induced damage in HBMVECs and regulates cell-to-cell contact, thus increasing barrier formation and maintenance.

### 3.5. Activation of STAT3 and ERK Is Important in the Function of CXCR4/SDF-1

**Figure 6:**
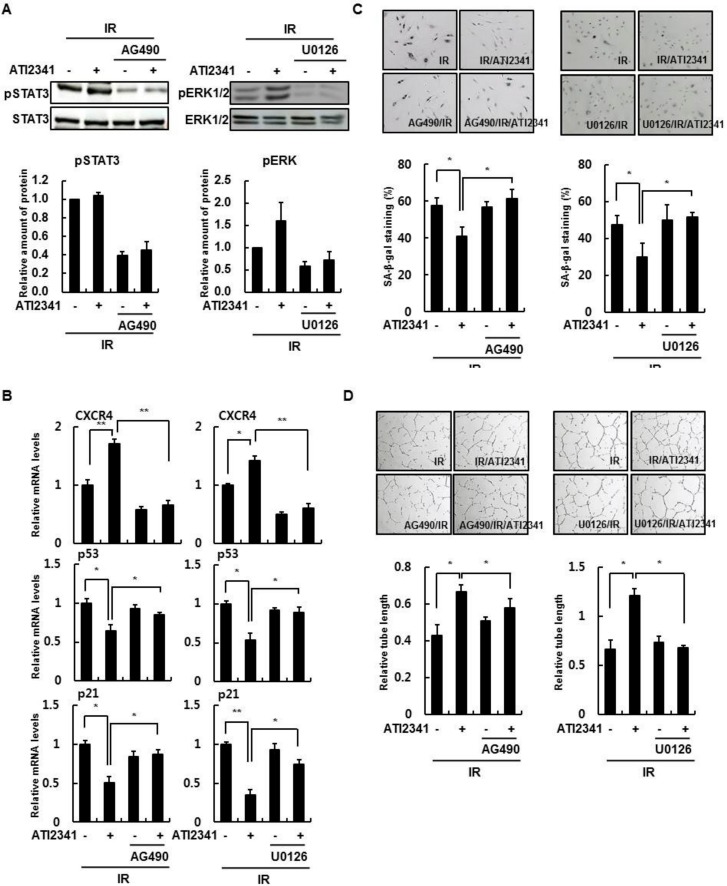
Phosphorylation of STAT3 and ERK regulates CXCR4 action. (**A**) HBMVECs were treated with 1 μM/mL AG490, a STAT3 inhibitor, or 1 μM/mL U0126, an ERK inhibitor. The cells were pretreated 1 μg/mL ATI2341, a CXCR4 agonist, 1 h before irradiation (4 Gy) and incubated for 24 h. Protein levels were confirmed by Western blot analysis. (**B**) Gene expression was measured by real-time polymerase chain reaction analysis. (**C**) After irradiation (4 Gy), cells were cultured for 4 days, and SA-β-gal positive cells were counted by SA-β-gal staining (×100). (**D**) Tube formation experiments were performed in STAT3- or ERK1/2-inhibited groups and ATI2341-treated groups. Values are expressed as the mean ± standard deviation of three independent experiments. * *p* < 0.05 and ** *p* < 0.01.

When cells treated with SDF-1 were irradiated, the increased phospho-p53, p53, and p21 levels decreased. However, when ERK and STAT3 activity was inhibited, SDF-1 function was not apparent (Figure 6A). In addition, when observed through real-time PCR, CXCR4 expression did not increase with SDF-1 treatment by inhibiting ERK and STAT3 activity. Therefore, the expression of p53 and p21 normally increased by senescence was not reduced by SDF-1 treatment (Figure 6B). In the SA-β-gal assay, irradiation increased activity, and SDF-1 treatment decreased activity. However, as expected, inhibition of ERK and STAT3 activity did not decrease SA-β-gal activity even after SDF-1 treatment (Figure 6C). Tube-forming was reduced by irradiation but was increased by SDF-1 treatment. However, inhibiting ERK and STAT3 activity did not result in increased tube-forming (Figure 6D). Hence, activation of ERK and STAT3 plays an important role in the regeneration of senescent cells along with SDF-1 treatment.

### 3.6. CXCR4 Agonist Mediates Radioprotection in Mice

**Figure 7:**
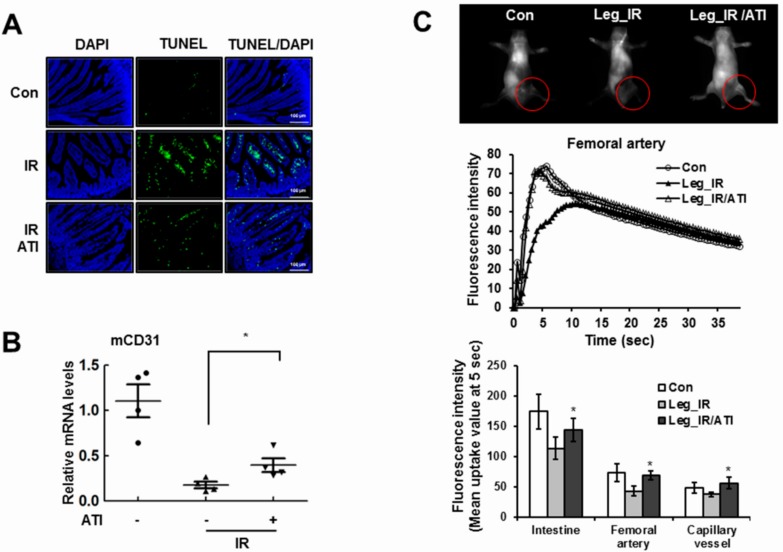
CXCR4 agonist effectively prevents radiation-induced damage in vivo. (**A**) In the small intestine of mice, apoptosis and ATI2341 levels were measured by a TUNEL assay; scale bars = 100 μm. (**B**) Mouse brains were dissected, and mRNA expression levels of *CD31*, a vascular endothelial cell marker, were determined in the cortex. (**C**) Maestro^TM^ equipment was used to observe the blood flow of the intestines, arteries, and microvessels after local radiotherapy under the left side. Values are expressed as the mean ± standard deviation of three independent experiments. * *p* < 0.05. TUNEL, terminal deoxynucleotidyl transferase dUTP nick end labeling.

Apoptosis in the intestinal villi was increased by radiation, but ATI2341 treatment decreased cell apoptosis (Figure 7A). The villi structure collapsed due to the damage, but the degree of collapse was reduced by ATI2341 treatment. The expression of *CD31*, measured for vascular endothelial cell density, indicated that endothelial cell loss in the cortex was induced by radiation treatment, and partially inhibited with an ATI2341 injection (Figure 7B). The effect of ATI2341 treatment alone in normal mice showed no difference compared to the control group (not shown). Abnormal anatomical or physiological vessel conditions caused by radiation are likely to alter time courses of a contrast agent following injection, compared with normal animals. As a result of measuring the circulation of the fluorescent dye, the initial blood flow of irradiated mice was slowed compared with normal mice, while ATI2341 treatment recovered radiation-induced blood flow rate decreases (Figure 7C). These results suggest that the CXCR4 agonist ATI2341 is involved in protecting vascular endothelial cells against DNA damaging agents, such as radiation in vivo, and alleviating tissue damage.

### 3.7. A CXCR4 Agonist Specifically Protects Mouse Brain Vessels and Contributes to the Improvement of Cognitive Disorders

**Figure 8:**
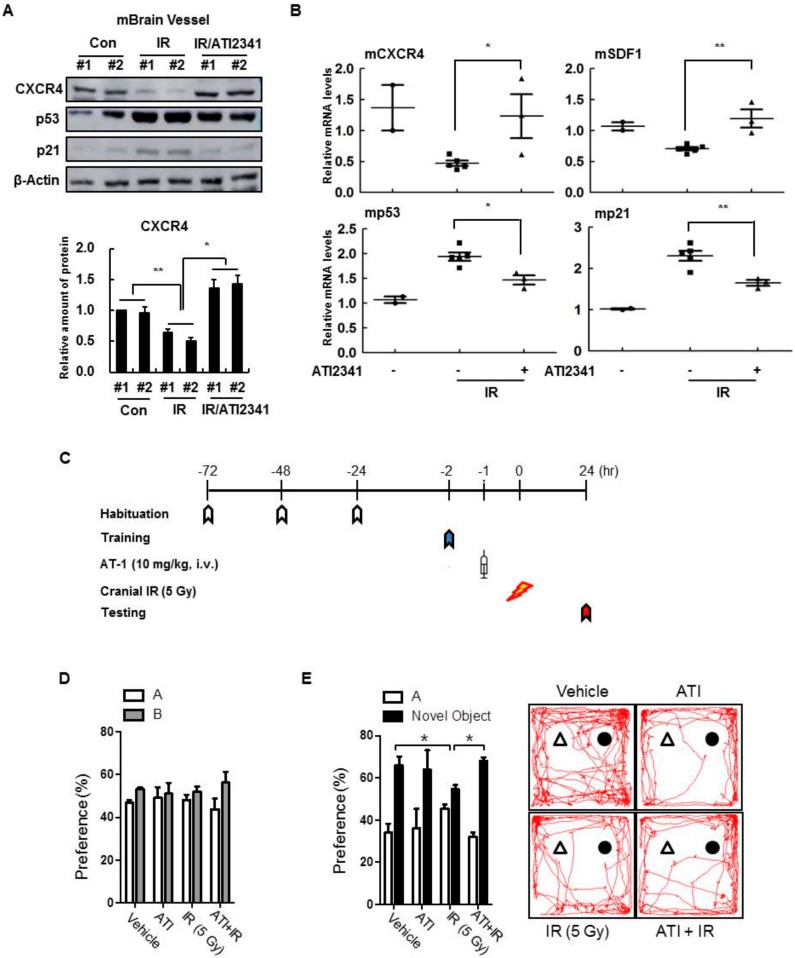
CXCR4 agonist specifically protects mouse brain vessels and restores cognitive activity. (**A**) Cerebral blood vessels were separated from mouse brains, and protein levels in the vessels were measured by Western blot analysis. (**B**) mRNA levels of *CXCR4, SDF-1, p53,* and *p21* were determined in the cerebral blood vessels. Values are expressed as the mean ± standard deviation of three independent experiments. * *p* < 0.05 and ** *p* < 0.01. (**C**) Schematic diagram illustrating the study protocol. The sham-irradiated mice and irradiated mice with or without ATI2341 were examined (*n* = 6 per group). During training, two objects were presented to each mouse for 10 min. After 24 h, one of the previous objects was replaced with a novel object (testing). (**D**) The mice of all groups before irradiation showed an equal preference for the two objects during training. (**E**) During testing, the control and ATI2341 treated groups exhibited significant preferences for the novel object. Mice following radiation and vehicle administration showed significant interrupted memory regarding the novel object, and a significant difference in novel object preference was found between and vehicle- and ATI-administration before cranial irradiation. Data represented as mean ± standard error. * *p* < 0.05.

Since a CXCR4 agonist could protect endothelial cells from death and blood vessel leakage from radiation exposure, we aimed to confirm the protective effect of CXCR4/SDF-1 on brain damage in vivo. The expression of CXCR4 by radiation was confirmed, but changes in CXCR4 expression were not observed in the hippocampus, cortex, or forebrain (not shown). Therefore, CD31 expression increased by ATI2341, and the effects of ATI2341 were specific only to blood vessels in brain tissue. By isolating blood vessels throughout the brain, it was confirmed that CXCR4 expression was reduced by radiation as well as restored by ATI2341 (Figure 8A). Conversely, ATI2341 injections decreased the expression of p53 and p21, which were increased by the injury. Similarly, Western blot analysis showed that ATI2341 increased *CXCR4* expression and decreased *p53* and *p21* expression (Figure 8B). Therefore, this study confirmed that ATI2341 increased CXCR4/SDF-1 expression and tissue damage restoration, specifically in cerebral vascular cells, and that these effects influenced cognitive ability. Object recognition experiments, conducted to measure abnormalities caused by cerebral vascular injury (Figure 8C), revealed that cranial irradiation in mice decreased object recognition memory, and ATI2341-injected mice had fewer abnormalities caused by radiation (Figure 8D,E). Thus, it was confirmed that CXCR4/SDF-1 alleviates blood vessel leakage and abnormal cognitive abilities caused by radiation. Together, CXCR4/SDF-1 restored normal cognitive ability and confirmed that improvements were caused by the function of ATI2341 specific to brain blood vessels.

## 4. Discussion

As the older population progressively increases, currently incurable neurodegenerative diseases are likely to have a devastating impact on individuals, families, and society. Despite the continuing the effort to understand the cause, mechanisms, and progress of these diseases, no effective or specific cures are available yet. Recently, vascular and inflammatory processes have been shown to contribute to the progress of many neurological diseases, specifically cerebral small vessel disease [28,29]. Cerebral small vessel disease, a term used for different pathological processes that affect the small vessels of the brain, is a leading cause of stroke [30], dementia [31], and gait problems [32] in elderly patients. An important pathophysiological mechanism of cerebral small vessel disease is endothelium dysfunction and reduces the appearance of tight junctions (TJs) and adherens junctions (AJs), efflux transporter proteins important for cerebrovascular protection, leading to blood–brain barrier leakage [33]; therefore, we investigated a potential approach to identify the disease through circulating biological factors, such as SDF-1, a ligand of CXCR4.

Radiotherapy and chemotherapy treatments cause diverse side effects in certain parts of the body, particularly in the cardiovascular and intestinal tract. Acute radiation effects are triggered by endothelial cell apoptosis, and chronic effects reflect cellular senescence in coronary arteries, cerebral circulation, and other microvascular systems [34]. Furthermore, radiation exposure is associated with an increased risk of aging-related neurodegenerative diseases. In the brain, endothelial cell injuries contribute to a systemic chronic inflammatory state that disturbs the cascade of normal aging processes and leads to the acceleration of age-related disorders, such as cognitive decline and dementia [2]. From in vitro and in vivo experiments, we provided knowledge of molecular mechanisms and consequences of radiation-induced damage to cerebrovascular endothelial cells. In the present study, radiation and anticancer drugs induced endothelial cell death and senescence in the brain, consistent with previous studies that show they can induce senescence in endothelial cells of various origins. 

This study shows that the downregulation of CXCR4/SDF-1 is associated with the induction of senescence (Figure 1 and Figure 2), and CXCR4 activation is vital for reducing genotoxic damage-induced senescence in vitro and in vivo. Notably, our results show that CXCR4 and SDF-1 expression decreased the induction of cellular senescence by radiation and anticancer drugs, evident by an increase in SA-β-gal activity and expression of p53 and p21 (Figure 3). These results show, for the first time, that CXCR4/SDF-1 is essential in the recovery of HBMVECs from radiation-induced cell damage. CXCR4/SDF-1 can also potentially promote neurogenesis to prevent local damage to the antineuronal area after brain ischemia. Under these conditions, the CXCR4/SDF-1 signaling pathway promotes neural progenitor migration around the injury site. Accordingly, CXCR4/SDF-1 promotes angiogenesis, improves cerebral neurogenesis, and facilitates cerebral vascular regrowth after a stroke. Our results also indicate that CXCR4/SDF-1 restored blood vessel damage and vascular leakage due to radiation, thus reducing blood flow function abnormalities and ameliorating cognitive abilities (Figure 8). 

CXCR7, another receptor for SDF-1, is expressed in human endothelial cells as well as CXCR4 [35,36,37,38]. Previous reports have shown that it has a 10-fold higher binding capacity than CXCR4 [39]. In addition, CXCR7 is known to play an important role in the development of the cardiovascular system in animal models, and cell adhesion, invasion, and blood vessel formation in vitro and in vivo are known to be enhanced by increasing CXCR7 [40,41,42,43]. Thus, we will further examine the effects of CXCR7 on cerebrovascular cells and animal models compared with CXCR4.

Several studies have aimed to improve and restore endothelial dysfunction in other pathological conditions [44]. One approach regarding the activation of nitric oxide signaling that regulates cerebral flow identified a protein marker for endothelial dysfunction [45]. As evidence of endothelial failure began to emerge in the form of elevated levels of markers of endothelial dysfunction, circulating biomarkers have also been considered in endothelial dysfunction studies. Studies using biomarkers of a cell-specific injury within the vasculature are needed to access vascular function information related to cognition and brain function, so it is possible to determine whether vascular dysfunction can precede neuronal dysfunction and cognitive impairments due to aging or during radiation exposure. Several individual circulating factors, such as growth differentiation factor 1 [13], gonadotropin-releasing hormone 1 [46], and insulin-like growth factor 2 [22], have positive actions but decrease with aging; they also increase neurogenesis and improve cognitive and memory function. Likewise, SDF-1 decreases in aged vessels, so SDF-1 therapy might be beneficial for brain vasculature as well as an aged brain overall.

Thus, our results show that CXCR4/SDF1 can inhibit cellular senescence and protect endothelial cells from external stress, including DNA-damaging agents, both in vitro and in vivo. Therefore, a CXCR4 agonist can be used to ameliorate cell damage and can be incorporated into a therapy that has the potential to treat diseases caused by DNA damage due to radiation therapy and chemotherapy.

## Figures and Tables

**Table 1:** Primers for real-time PCR analysis.

Gene	Forward Primer	Reverse Primer
*hGAPDH*	GACCACAGTCCATGCCATCA	GTCAAAGGTGGAGGAGTGGG
*hCXCR4*	CACCGAGGCCCTAGCTTTCT	CACAGAGGTGAGTGCGTGCT
*hSDF1*	CAGATGCCCATGCCGATT	AGTTTGGAGTGTTGAGAATT
*hp53*	GAACAAGTTGGCCTGCACTG	GAAGTGGGCCCCTACCTAGA
*hp21*	TATGGGGCTGGGAGTAGTTG	CGAGAGAAAACAGTCCAGGC
*hp16*	AGTCCTCCTTCCTTGCCAAC	TCCGAGCACTTAGCGAATGT
*hNBS1*	CCACCATTGTCCTAGCTACT	CTTGACTGGAACTCCCTTCT
*hBRCA1*	GTGGTGCTTCTGTGGTGAAG	ACAGGTGCCTCACACATCTG
*h53BP1*	AGGTTGGGTGTTCTTTGGCTT	TTGGTGTTGAGGCTTGTGGT
*hChk1*	CTTTGGCTTGGCAACAGT	CCAGTCAGAATACTCCTG
*hChk2*	GCGCCTGAAGTTCTTGTTTC	GCCTTTGGATCCACTACCAA
*mGAPDH*	TCAACGACCCCTTCATTGAC	ATGCAGGGATGATGTTCTGG
*mCXCR4*	TCAGTGGCTGACCTCCTCTT	TTTCAGCCAGCAGTTTCCTT
*mSDF1*	GGTTCTTCGAGAGCCACATC	GGGCAGCCTTTCTCTTCTTC
*mp53*	CTCCGAAGACTGGATGACTGC	CAACAGATCGTCCATGCAGTG
*mp21*	ACGGTGGAACTTTGACTTCGTC	CAGAGTGCAAGACAGCGACAAG
*mCD31*	AGGCTTGCATAGAGCTCCAG	TTCTTGGTTTCCAGCTATGG

## Supplementary Materials

The following are available online at https://www.mdpi.com/2073-4409/8/10/1230/s1, Figure S1: Cell proliferation effect of SDF1 and ATI2341 on HBMVECs. Figure S2: Graph of density measurement of bands in Western blots.
