# Supplementary material for: Stromal Cell-Derived Factor 1 Protects Brain Vascular Endothelial Cells from Radiation-Induced Brain Damage
Source: Cells. 2019 Oct 10;8(10):1230. doi: 10.3390/cells8101230 (PMC6830118; doi:10.3390/cells8101230)
Supplement: Supplementary file 1 [file cells-08-01230-s001.pdf]

Supplementary Data

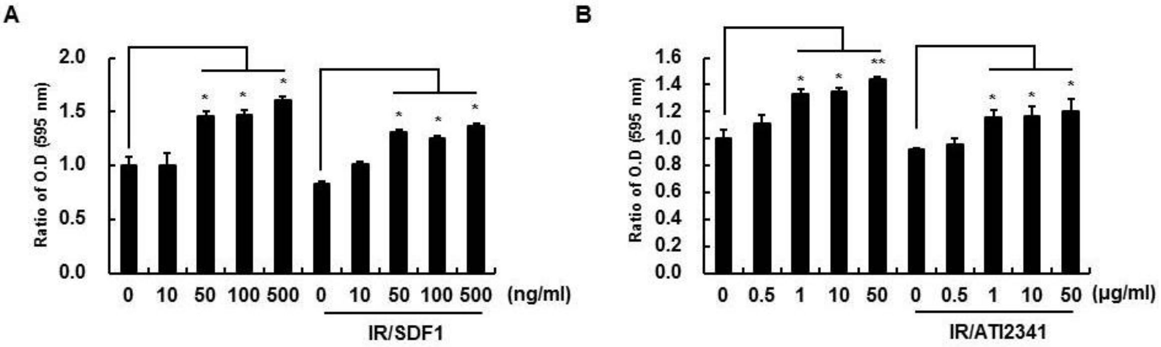

Figure S1. Cell proliferation effect of SDF1 and ATI2341 on HBMVECs. (A) SDF1 was treated at a concentration of 10 – 500 ng/ml. After reacting for 2 days, cell proliferation was measured using MTT assay. (B) ATI2341 was treated at a concentration of 0.5 – 50 μg/ml. After reacting for 2 days, cell proliferation was measured using MTT assay. The radiation was treated at a concentration of 4 Gy. Values are expressed as the mean ± standard deviation of 3 independent experiments. *P<0.05 and **P<0.01.

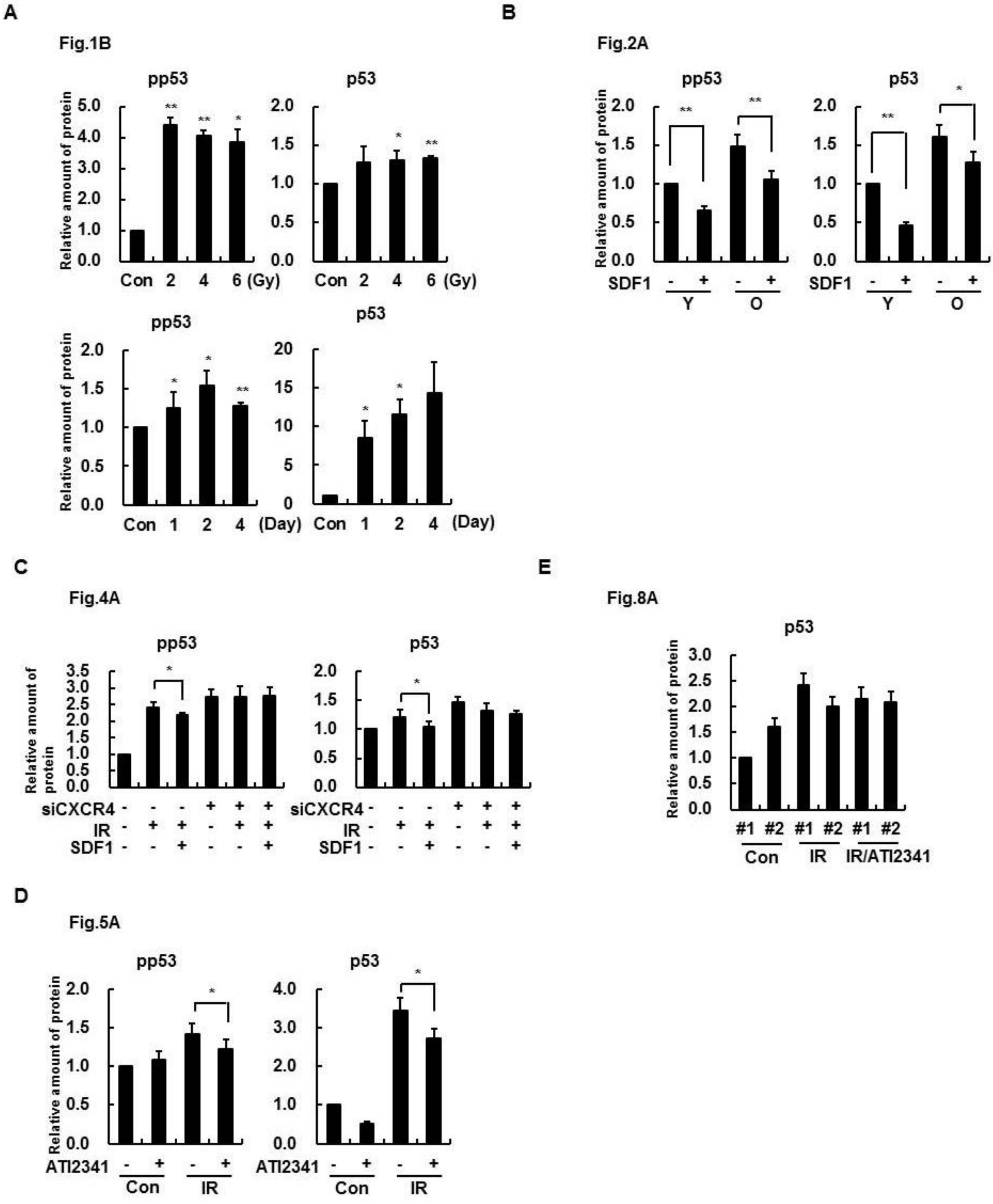

Figure S2. Graph of density measurement of bands in Western blots. Values are expressed as the mean ± standard deviation of 3 independent experiments. *P<0.05 and **P<0.01.
